# Pituitary Adenylate Cyclase-Activating Polypeptide—A Neuropeptide as Novel Treatment Option for Subacute Ileitis in Mice Harboring a Human Gut Microbiota

**DOI:** 10.3389/fimmu.2019.00554

**Published:** 2019-03-22

**Authors:** Stefan Bereswill, Ulrike Escher, Anne Grunau, Anja A. Kühl, Ildiko R. Dunay, Andrea Tamas, Dora Reglodi, Markus M. Heimesaat

**Affiliations:** ^1^Department of Microbiology, Infectious Diseases, and Immunology, Charité—Universitätsmedizin Berlin, Corporate Member of Freie Universität Berlin, Humboldt-Universität zu Berlin, and Berlin Institute of Health, Berlin, Germany; ^2^Department of Medicine I for Gastroenterology, Infectious Diseases and Rheumatology/Research Center ImmunoSciences (RCIS), Charité—Universitätsmedizin Berlin, Corporate Member of Freie Universität Berlin, Humboldt-Universität zu Berlin, and Berlin Institute of Health, Berlin, Germany; ^3^Medical Faculty, Institute of Inflammation and Neurodegeneration, University Hospital Magdeburg, Magdeburg, Germany; ^4^Department of Anatomy, MTA-PTE PACAP Research Team, Centre for Neuroscience, University of Pecs Medical School, Pecs, Hungary

**Keywords:** pituitary adenylate cyclase-activating polypeptide (PACAP), subacute ileitis, Th1-type immunopathology, human fecal microbiota transplantation, gut-brain axis, preclinical intervention study

## Abstract

The neuropeptide Pituitary adenylate cyclase-activating polypeptide (PACAP) is well-known for its important functions in immunity and inflammation. Data regarding anti-inflammatory properties of PACAP in the intestinal tract are limited, however. In our present preclinical intervention study we addressed whether PACAP treatment could alleviate experimental subacute ileitis mimicking human gut microbiota conditions. Therefore, secondary abioitic mice were subjected to human fecal microbiota transplantation (FMT) and perorally infected with low-dose *Toxoplasma gondii* to induce subacute ileitis on day 0. From day 3 until day 8 post-infection, mice were either treated with synthetic PACAP38 or placebo. At day 9 post-infection, placebo, but not PACAP treated mice exhibited overt macroscopic sequelae of intestinal immunopathology. PACAP treatment further resulted in less distinct apoptotic responses in ileal and colonic epithelia that were accompanied by lower T cell numbers in the mucosa and lamina propria and less secretion of pro-inflammatory cytokines in intestinal *ex vivo* biopsies. Notably, ileitis-associated gut microbiota shifts were less distinct in PACAP as compared to placebo treated mice. Inflammation-ameliorating effects of PACAP were not restricted to the intestines, but could also be observed in extra-intestinal including systemic compartments as indicated by lower apoptotic cell counts and less pro-inflammatory cytokine secretion in liver and lungs taken from PACAP treated as compared to placebo control mice, which also held true for markedly lower serum TNF and IL-6 concentrations in the former as compared to the latter. Our preclinical intervention study provides strong evidence that synthetic PACAP alleviates subacute ileitis and extra-intestinal including systemic sequelae of T cell-driven immunopathology. These findings further support PACAP as a novel treatment option for intestinal inflammation including inflammatory bowel diseases (IBD).

## Introduction

The Pituitary adenylate cyclase-activating polypeptide (PACAP) could be first identified in the hypothalamus exerting adenylate cyclase stimulating activity within the pituitary gland (1). Being part of the vasoactive intestinal peptide (VIP)/secretin/glucagon family, the neuropeptide shares 68% sequence homology with VIP and presents with two biologically active amidated forms (i.e., PACAP 27 and PACAP38) after alternative splicing from its pre-pro precursor (1, 2). Beyond the nervous system, PACAP expression can be found in many peripheral organs within the reproductive, respiratory, endocrine and digestive system as well as in lymphoid compartments including immune cells (2). PACAP is able to bind to VPAC1, VPAC2, and PAC1 receptors on innate immune cells including macrophages and lymphocytes (3–5). Given its virtual ubiquitous expression, PACAP presents with a variety of cyto-protective properties including anti-inflammatory and anti-apoptotic effects (5, 6). In experimental models of encephalomyelitis and arthritis, for instance, distinct anti-inflammatory effects following exogenous PACAP application have been demonstrated (7, 8). Data regarding inflammation-ameliorating properties of synthetic PACAP in the gastrointestinal tract are limited, however. PACAP^−/−^ mice suffered from more severe acute colitis following dextran sodium sulfate (DSS) challenge as compared to wildtype counterparts (9, 10).

Human inflammatory bowel diseases (IBD) such as Crohn's disease and ulcerative colitis comprise chronic inflammatory conditions with acute episodes within the gastrointestinal tract and are of multi-factorial etiology (11–13). Most *in vivo* studies mimicking human IBD have applied experimental models of the large intestines so far, whereas, however, small intestinal inflammation models are rather scarce (14).

In our previous studies we applied an acute ileitis model characterized by a severe T cell-driven immunopathology with a lethal outcome within 1 week after peroral high-dose (i.e., >50 cysts) *Toxoplasma gondii* infection of mice (14–17). This high-dose *T. gondii* infection model mimics key features of the acute phase of human Crohn's disease (“ileitis terminalis”), given (i) the predilection site of the terminal ileum, (ii) the underlying T helper cell (Th)−1 immunopathology that is (iii) associated with marked shifts in gut microbiota composition (dysbiosis) toward Gram-negative gut commensals, (iv) further contributing to an acceleration of the inflammatory scenario via Toll-like receptor (TLR)−4 dependent signaling of lipopolysaccharide (LPS) derived from the Gram-negative commensal bacterial cell walls (14). We were able to show that treatment of mice with synthetic PACAP could efficiently ameliorate acute ileitis and even extra-intestinal sequelae of *T. gondii* infection in a time-of-treatment dependent fashion with highest efficacy during a prophylactic regimen when starting PACAP application prior ileitis induction (17). The within 1 week lethal outcome of the hyper-acute inflammatory scenario following high-dose *T. gondii* infection needs to be considered as a limitation of the applied gut inflammation model, however.

This prompted us to unravel potential immune-modulatory properties of PACAP during small intestinal inflammation of less acute severity. Since the host specific gut microbiota is known to be essentially involved in the onset, progress, and outcome of distinct immunopathological conditions including intestinal inflammation (15, 18, 19), we generated (with respect to their gut microbiota) “humanized” mice. Very recently we were able to show that within 9 days following peroral infection with a low-dose (i.e., 1 cyst) of *T. gondii*, mice harboring a human gut microbiota develop non-lethal subacute ileitis characterized by increased T cell-dependent gut epithelial apoptosis and pro-inflammatory cytokine secretion in intestinal and extra-intestinal compartments (20). Furthermore, low-dose *T. gondii* infected mice displayed rather mild-to-moderate histopathological changes of the ileal mucosa and lamina propria, whereas no transmural small intestinal necrosis like in the lethal high-dose infection model could be observed. In the present preclinical intervention study we assessed whether therapeutic PACAP application starting 3 days after ileitis induction (i) resulted in disease-alleviating effects in the intestinal tract, (ii) was associated with distinct shifts in gut microbiota composition, and furthermore, (iii) whether potential PACAP-induced anti-inflammatory effect could also be observed in extra-intestinal organs or (iv) even in systemic compartments.

## Materials and Methods

### Generation of Mice With a Human Gut Microbiota by Fecal Microbiota Transplantation

Female C57BL/6j mice were raised and maintained under specific pathogen-free (SPF) conditions in the Forschungseinrichtungen für Experimentelle Medizin (FEM, Charité - University Medicine, Berlin, Germany). Mice with a depleted microbiota (i.e., secondary abiotic mice) were generated as reported earlier (15, 18). Briefly, eight-week-old mice were kept in autoclaved cages and treated with an antibiotic cocktail for 8 weeks containing ampicillin plus sulbactam (1 g/L; Ratiopharm, Germany), vancomycin (500 mg/L; Cell Pharm, Germany), ciprofloxacin (200 mg/L; Bayer Vital, Germany), imipenem (250 mg/L; MSD, Germany) and metronidazole (1 g/L; Fresenius, Germany) (*ad libitum*). Successful depletion of the gut microbiota was confirmed in fecal samples by both, culture and molecular (16S rRNA based) methods as stated elsewhere (18, 21). In order to guarantee antibiotic washout, the antibiotic cocktail was replaced by sterile tap water (*ad libitum*) 3 days before human fecal microbiota transplantation (FMT). Fresh fecal samples that were negative for enteropathogenic bacteria, viruses, and parasites were donated from five healthy human individuals, dissolved in sterile phosphate buffered saline (PBS; Gibco, Life Technologies, United Kingdom) and stored at −80°C until usage as described previously (18). Immediately before FMT, individual fecal aliquots were thawed, pooled and applied to secondary abiotic mice by gavage on three consecutive days (18). Between individual FMT challenges, bacterial loads varied <0.5 orders of magnitude (Figure S1). For appropriate establishment of the complex human gut microbiota within the murine host, mice were kept for 12 days before subacute ileitis induction. Immediately before peroral *T. gondii* infection (d0) and at the end of the observation period [i.e., day 9 post-infection (p.i.)] individual fecal samples were collected from human microbiota associated (hma) mice for subsequent quantification of the main gut bacterial groups by molecular methods as described elsewhere (15, 18, 19).

### Induction of Subacute Ileitis, Determination of Parasitic Loads

For induction of subacute ileitis, hma mice were perorally challenged with one cyst of *T. gondii* strain ME49 in 0.3 mL brain suspension by gavage as reported earlier (20). *T gondii* DNA was quantitated in ileal *ex vivo* biopsies as stated previously (16).

### Treatment

PACAP38 was synthesized at the Department of Medical Chemistry, University of Szeged (Hungary) and applied to mice with a daily dose of 1.5 mg per kg body weight (in PBS) (9, 17). Mice received either the synthetic PACAP38 or PBS as placebo control (PLC) via the intraperitoneal (i.p.) route (0.3 mL) from day 3 p.i. until day 8 p.i. once daily.

### Clinical Conditions and Sampling Procedures

Clinical conditions of mice including body weight loss were monitored daily. Nine days post ileitis induction mice were sacrificed by isoflurane treatment (Abbott, Germany). Cardiac blood and *ex vivo* biopsies were derived from mesenteric lymph nodes (MLN), lung, liver, ileum and colon under aseptic conditions. Respective tissue samples were taken from each mouse in parallel for immunological, immunohistochemical, and microbiological analyses. Whereas the small intestinal lengths were assessed by measuring the distance from the duodenum leaving the stomach to the ileal-cecal valve, the colonic lengths were measured from the ileal-cecal valve to the rectum with a ruler and expressed in cm.

### Histopathology and Immunohistochemistry

*Ex vivo* biopsies were derived from the terminal ileum, colon, lung, and liver, fixed in 5% formalin and embedded in paraffin. Histopathological changes were quantitated in 5 μm thin, with hematoxylin and eosin (H&E) stained ileal paraffin sections applying a standardized histopathological scoring system ranging from 0 to 6 as stated elsewhere (15).

Paraffin sections (5 μm) were further analyzed applying *in situ* immunohistochemistry as reported previously (22). Briefly, in order to quantitate apoptotic cells and T lymphocytes, primary antibodies against cleaved caspase-3 (Asp175, #9661, Cell Signaling, Leiden, Netherlands; 1:200), and CD3 (#IR50361-2, Dako, Santa Clara, CA, USA; 1:5) were applied, respectively. The average number of positively stained cells (within at least six high power fields (HPF), 0.287 mm^2^, 400x magnification) was assessed by an independent and blinded investigator.

### Pro-inflammatory Cytokine Secretion

Ileal and colonic tissue samples (~1 cm^2^) were cut longitudinally and washed in PBS. Respective intestinal *ex vivo* biopsies as well as samples derived from MLN (3 lymph nodes), liver samples (~1 cm^2^) and lung were placed in 24-flat-bottom well-culture plates (Falcon, Germany) supplemented with 500 μL serum-free RPMI 1640 medium (Gibco, life technologies), penicillin (100 U/mL, Biochrom, Germany) and streptomycin (100 μg/mL; Biochrom). After overnight incubation at 37°C, culture supernatants were taken and tested for IL-6, TNF, and IFN-γ secretion applying the Mouse Inflammation Cytometric Bead Assay (CBA; BD Bioscience) on a BD FACSCanto II flow cytometer (BD Bioscience). Systemic pro-inflammatory cytokine concentrations were measured in serum samples.

### Molecular Analysis of the Human Fecal Donor Suspensions and the Intestinal Microbiota

Human fecal donor suspensions as well as fresh ileal and colonic luminal samples were immediately transferred to liquid nitrogen and stored at −80°C until further analyses. Fecal DNA extraction was performed as reported earlier (15). Briefly, the amount of DNA was assessed with a Quant-iT PicoGreen reagent (Invitrogen, UK) and adjusted to 1 ng per μL. The main human gut bacterial groups including enterobacteria, enterococci, lactobacilli, bifidobacteria, *Bacteroides/Prevotella* species, *Clostridium coccoides* group, and *Clostridium leptum* group as well as the total eubacterial loads were determined applying quantitative real-time polymerase chain reaction (qRT-PCR) and species-, genera- or group-specific 16S rRNA gene primers (Tib MolBiol, Germany) as indicated (Figure S2) and further described previously (19, 23) (expressed as 16S rRNA gene copies per ng DNA).

### Bacterial Translocation

In order to survey viable bacteria translocating from the gastrointestinal tract to extra-intestinal including systemic tissue sites, *ex vivo* biopsies were taken from MLN, lungs, and liver, homogenized in sterile PBS and analyzed in serial dilutions on defined solid culture media as reported previously (15, 24). Cardiac blood was incubated in thioglycolate enrichment broths (BD Bioscience, Germany) for at least 7 days at 37°C and then streaked on culture media (25, 26). For at least 2 days bacteria were grown under aerobic, microaerobic and anaerobic conditions at 37°C.

### Statistical Analysis

Medians and levels of significance were determined by the one-way ANOVA test followed by Tukey post-correction test for multiple comparisons. Two-sided probability (*p*) values ≤0.05 were considered significant. Experiments were reproduced three times.

## Results

### Macroscopic Sequelae in PACAP Treated Mice With a Human Gut Microbiota Suffering From Subacute Ileitis

**Figure 1:**
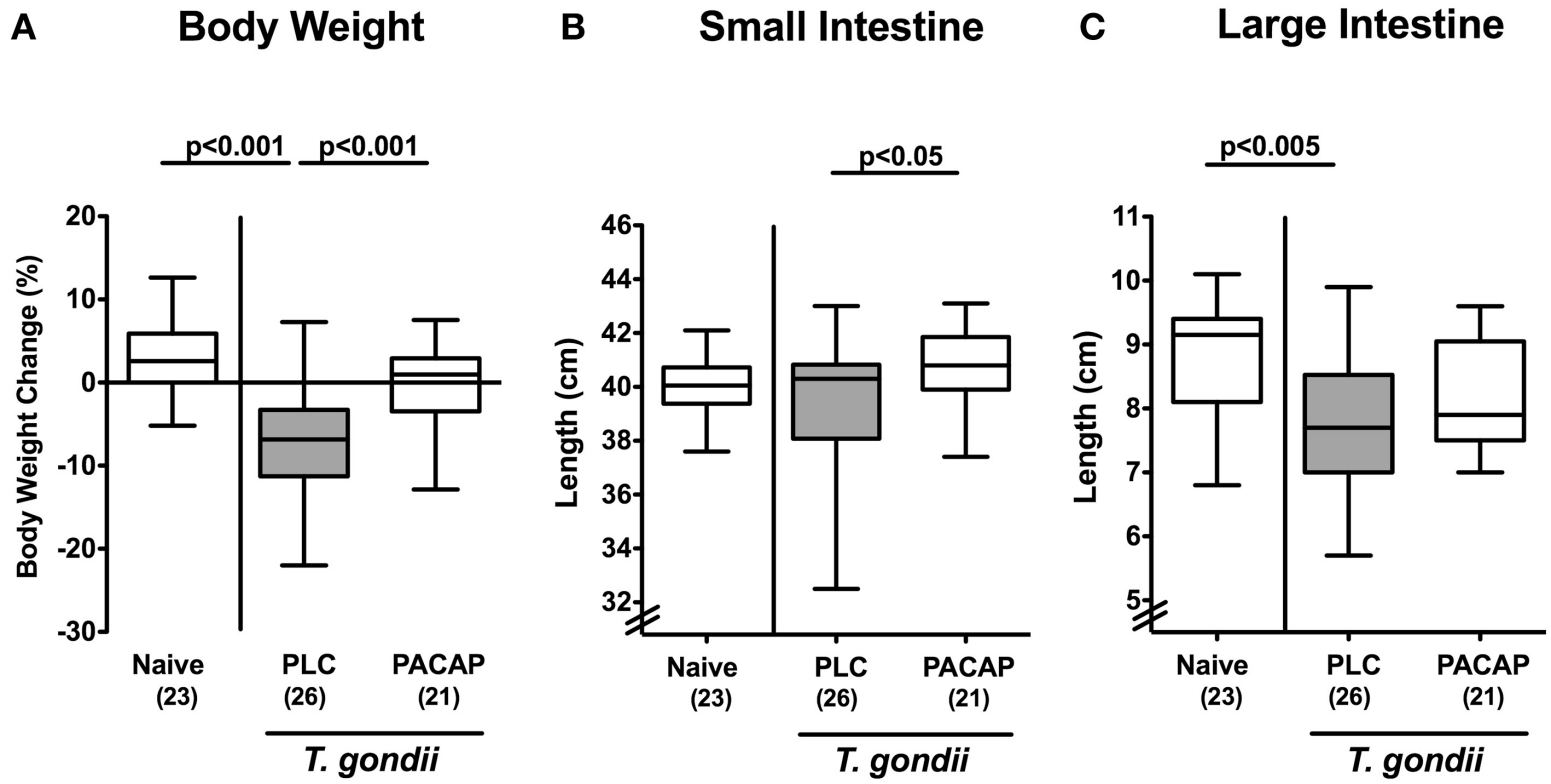
Macroscopic effects in PACAP treated mice with a human gut microbiota suffering from subacute ileitis. Subacute ileitis was induced by *T. gondii* infection of mice harboring a human gut microbiota (day 0). Starting 3 days post-infection (p.i.), mice were either treated with PACAP or placebo (PLC). Uninfected mice with a human microbiota served as control animals (Naive). At day 9 p.i., **(A)** relative body weight loss, **(B)** small intestinal, and **(C)** large intestinal lengths were assessed. Box plots represent the 75 and 25th percentiles of the median (black bar inside the boxes). The total range and significance levels determined by one-way ANOVA test followed by Tukey post-correction test for multiple comparisons are shown. Total numbers of analyzed animals are given in parentheses. Data were pooled from four independent experiments.

In the present preclinical intervention study we addressed whether therapeutic application of synthetic PACAP dampened pro-inflammatory responses in intestinal and extra-intestinal including systemic compartments of mice with a human gut microbiota suffering from subacute ileitis. The small intestinal immunopathology was induced by peroral low dose *T. gondii* infection on day 0 (20). Three days post ileitis induction, hma mice were treated with synthetic PACAP for 6 days in total and clinical conditions including the body weights were monitored. Remarkably, *T. gondii* infected placebo (PLC) control mice exhibited substantial body weight loss until day 9 p.i., whereas this was not the case in PACAP treated mice (Figure 1A). Given that intestinal inflammation is accompanied by a significant shortening of the inflamed intestine (15, 18, 27), we measured small intestinal lengths upon necropsy. At day 9 p.i., PACAP treated mice displayed slightly longer small intestines as compared to PLC control animals (Figure 1B). Of note, the standard deviation within the PLC group was relatively high, which might explain the non-significant differences compared to the naive cohort (Figure 1B). Even though the terminal ileum is well-known to be the predilection site of *T. gondii* induced immunopathology (14), we also assessed the lengths of the large intestines. Interestingly, PLC, but not PACAP treated mice displayed significantly shorter large intestines at day 9 p.i. as compared to naive mice (Figure 1C), pointing toward inflammatory responses beyond the terminal small intestine, additionally affecting the large intestinal tract. Hence, PACAP treatment resulted in a better clinical / macroscopic outcome of *T. gondii* induced subacute ileitis. To rule out that the observed differences in disease outcomes might be due to different *T. gondii* infection efficiencies, we assessed parasitic DNA concentrations in ileal *ex vivo* biopsies at day 9 p.i., but detected comparable ileal *T. gondii* loads in mice of the PACAP and PLC cohort (not shown).

### Intestinal Anti-inflammatory Effects in PACAP Treated Mice With a Human Gut Microbiota Suffering From Subacute Ileitis

**Figure 2:**
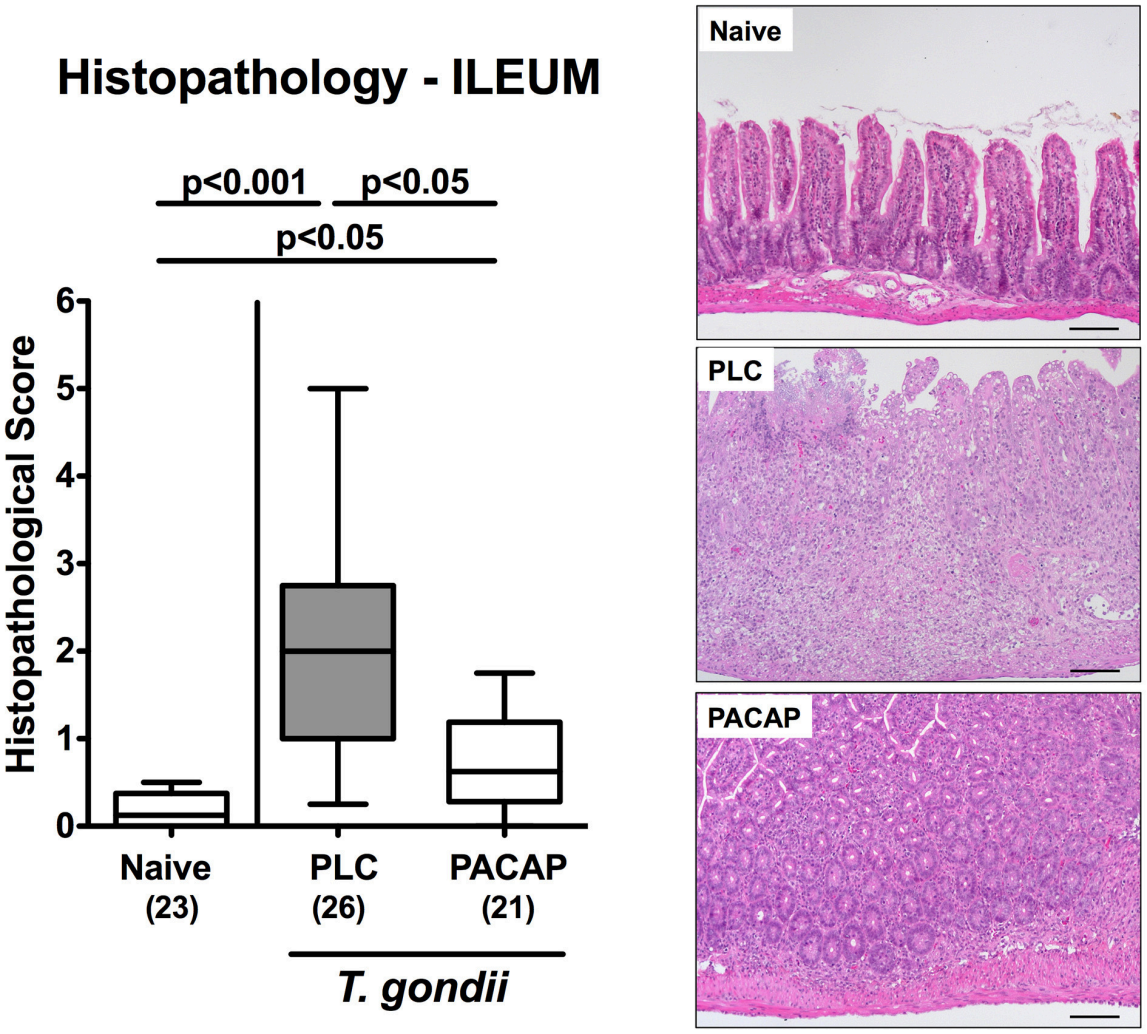
Microscopic effects in PACAP treated mice with a human gut microbiota suffering from subacute ileitis. Subacute ileitis was induced by *T. gondii* infection of mice harboring a human gut microbiota (day 0). Starting 3 days post-infection (p.i.), mice were either treated with PACAP or placebo (PLC). Uninfected mice with a human microbiota served as control animals (Naive). At day 9 p.i., histopathological changes within the ileum were quantitated in H&E stained ileal paraffin sections applying a standardized histopathological scoring system (left). Box plots represent the 75 and 25th percentiles of the median (black bar inside the boxes). The total range and significance levels determined by one-way ANOVA test followed by Tukey post-correction test for multiple comparisons are shown. Total numbers of analyzed animals are given in parentheses. Data were pooled from four independent experiments. Histopathological changes are illustrated in photomicrographs representative for four independent experiments (right; 100x magnification, scale bar 100 μm).

**Figure 3:**
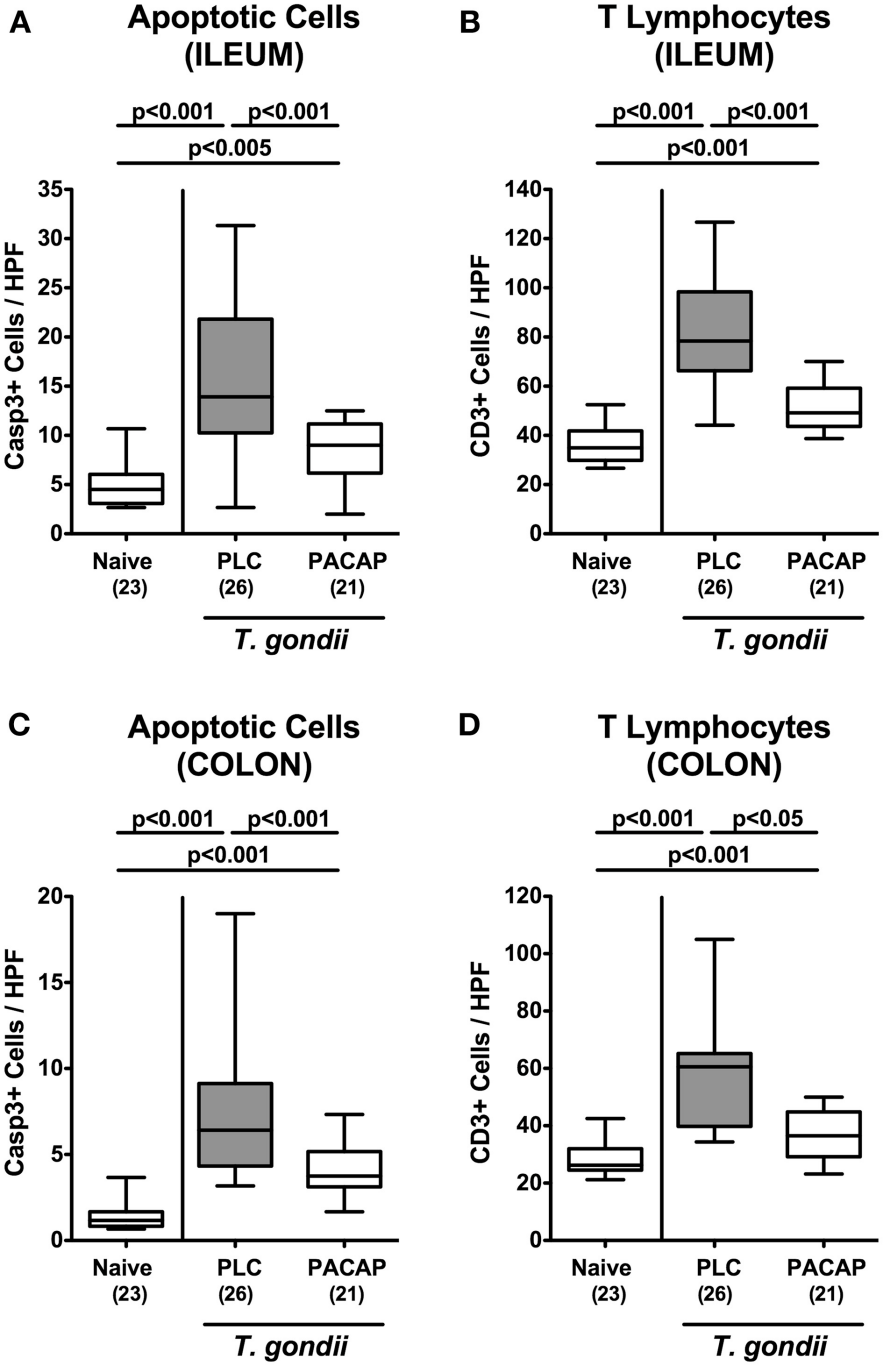
Apoptotic epithelial cell and T lymphocyte responses in the intestinal tract following PACAP treatment of mice with a human gut microbiota suffering from subacute ileitis. Subacute ileitis was induced by *T. gondii* infection of mice harboring a human gut microbiota (day 0). Starting 3 days post-infection (p.i.), mice were either treated with PACAP or placebo (PLC). Uninfected mice with a human microbiota served as control animals (Naive). At day 9 p.i., the average numbers of apoptotic epithelial cells [Casp3+; **(A,C)**] and of T lymphocytes [CD3+; **(B,D)**] in at least six high power fields (HPF) were quantitatively assessed in ileal **(A,B)** and colonic **(C,D)** paraffin sections applying *in situ* immunhistochemistry. Box plots represent the 75 and 25th percentiles of the median (black bar inside the boxes). The total range and significance levels determined by one-way ANOVA test followed by Tukey post-correction test for multiple comparisons are shown. Total numbers of analyzed animals are given in parentheses. Data were pooled from four independent experiments.

We next addressed PACAP-induced anti-inflammatory effects in the intestinal tract on microscopic level. At day 9 following ileitis induction ileal histopathological scores were lower in PACAP treated hma mice as compared to PLC control animals (Figure 2). Of note, we could not observe significant differences in histopathological changes in the mucosa and lamina propria of H&E stained colonic paraffin sections (not shown). Since apoptosis is regarded a reliable parameter for the histopathological grading of intestinal inflammation (16), we further assessed *T. gondii* induced apoptotic cell responses in the intestines by quantification of caspase3+ intestinal epithelial cells applying *in situ* immunohistochemistry. At day 9 p.i., hma mice displayed multifold increased numbers of apoptotic ileal epithelial cells (Figure 3A). These increases were, however, less pronounced in PACAP treated mice (Figure 3A; Figure S3A).

Given that T lymphocytes are the major driving forces of *T. gondii* induced ileitis (14), we additionally stained ileal paraffin sections with CD3 antibodies. Ileitis induction was, in fact, accompanied by a marked increase in CD3+ ileal epithelial cell numbers until day 9 p.i. (Figure 3B, Figure S3B), but to a significantly lesser extent upon PACAP treatment (Figure 3B, Figure S3B). Remarkably, *T. gondii*-induced increases in both, caspase3+ and CD3+ cells could also be observed in the epithelia and mucosa / lamina propria of the large intestines, respectively (Figures 3C,D, Figures S3C,D). Like in the ileal compartment, PACAP treatment was accompanied with significantly less distinct apoptosis and abundance of T lymphocytes in the large intestinal tract (Figures 3C,D, Figures S3C,D).

**Figure 4:**
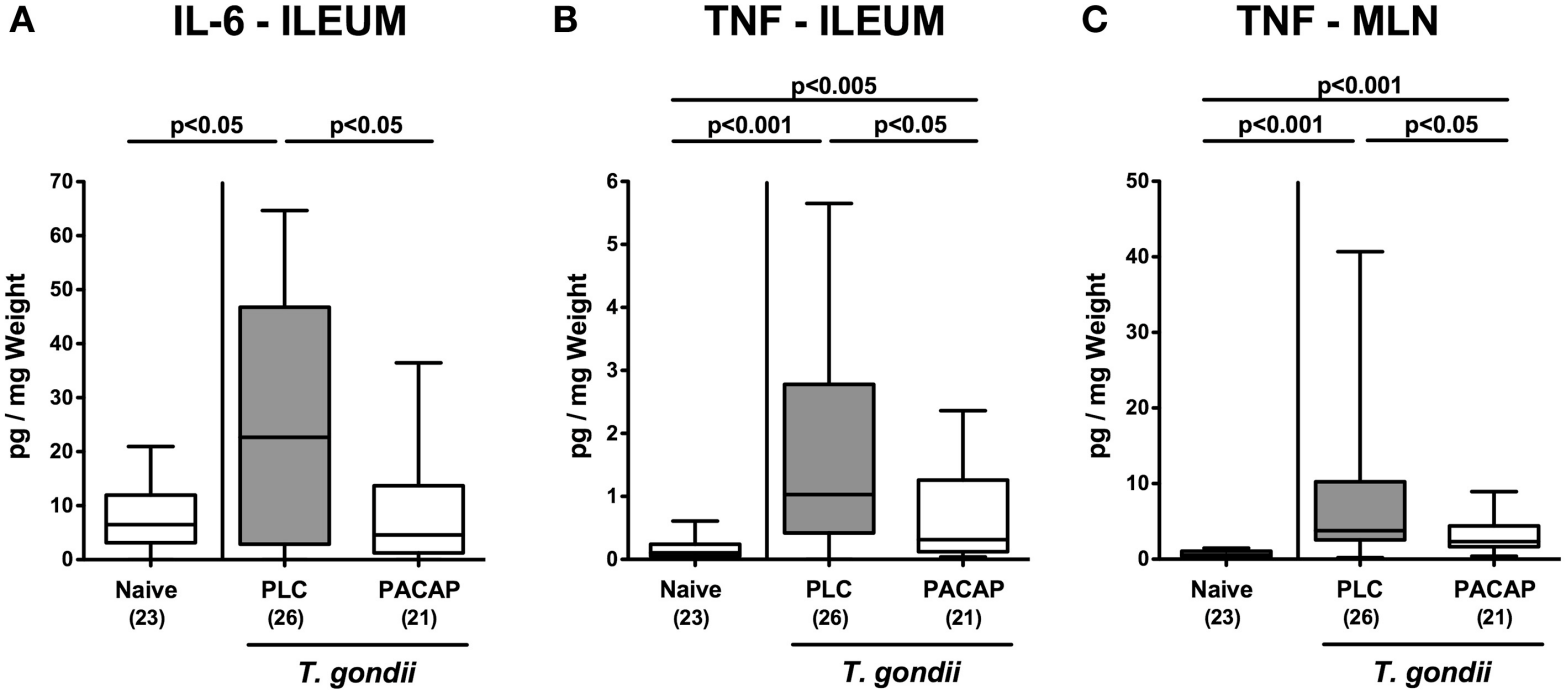
Intestinal pro-inflammatory cytokine secretion upon PACAP treatment of mice with a human gut microbiota suffering from subacute ileitis. Subacute ileitis was induced by *T. gondii* infection of mice harboring a human gut microbiota (day 0). Starting 3 days post-infection (p.i.), mice were either treated with PACAP or placebo (PLC). Uninfected mice with a human microbiota served as control animals (Naive). At day 9 p.i., secretion of pro-inflammatory cytokines such as IL-6 **(A)** and TNF **(B,C)** were assessed in *ex vivo* biopsies derived from the ileum **(A,B)** and from mesenteric lymph nodes [MLN; **(C)**]. Box plots represent the 75 and 25th percentiles of the median (black bar inside the boxes). The total range and significance levels determined by one-way ANOVA test followed by Tukey post-correction test for multiple comparisons are shown. Total numbers of analyzed animals are given in parentheses. Data were pooled from four independent experiments.

We further assessed pro-inflammatory cytokine secretion in intestinal *ex vivo* biopsies. At day 9 p.i., PLC, but not PACAP treated mice exhibited higher IL-6 concentrations in their ileum as compared to naive counterparts (Figure 4A). In addition, TNF secretion was far less pronounced in the ileum and MLN of mice from the PACAP cohort as compared to PLC control animals (Figures 4B,C). Hence, PACAP exerts potent inflammation-alleviating effects in the intestinal tract of hma mice during subacute ileitis that are not restricted to the terminal ileum.

### Changes in Gut Microbiota Composition in PACAP Treated Mice With a Human Gut Microbiota Suffering From Subacute Ileitis

**Figure 5:**
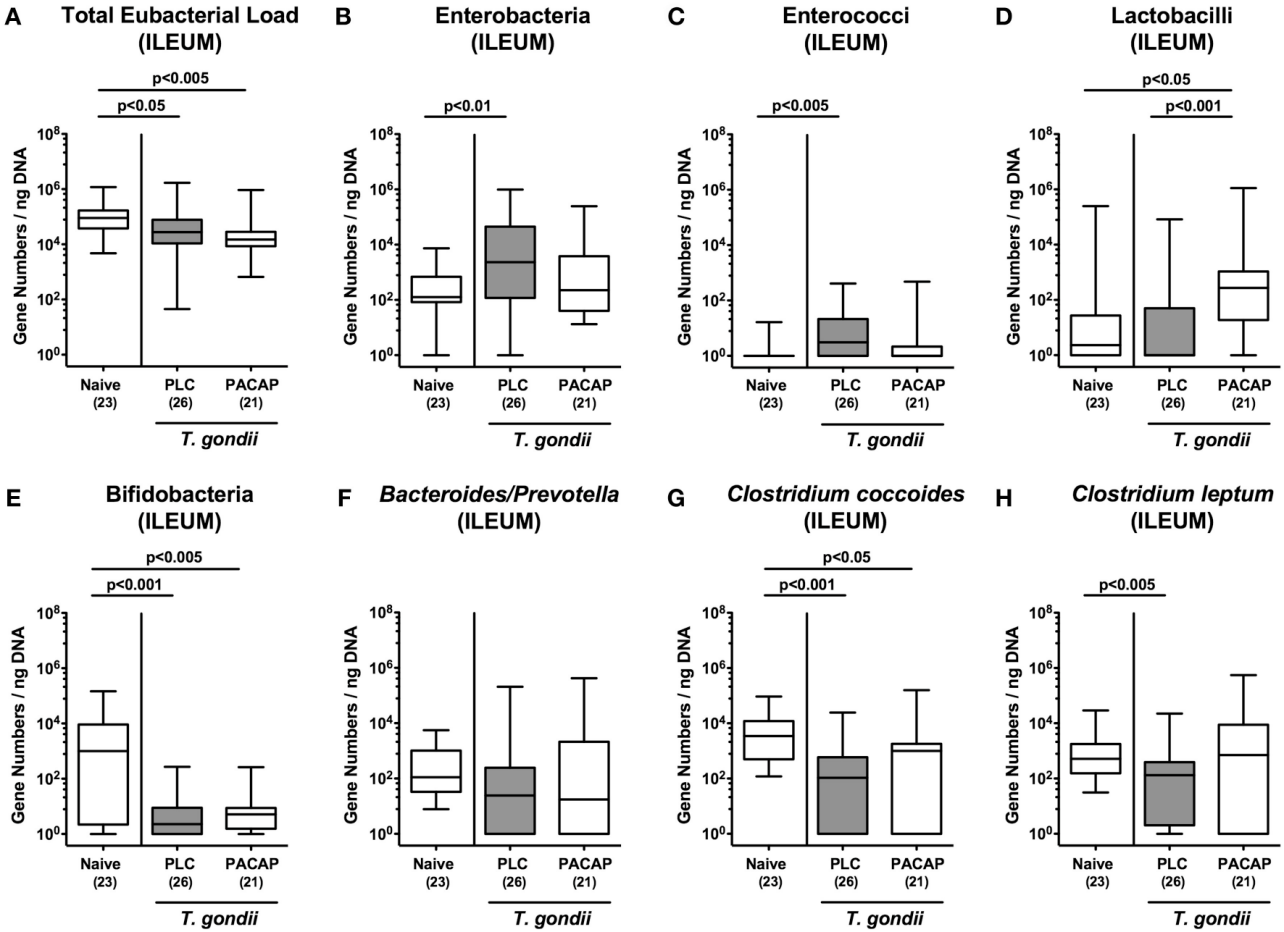
Colonic microbiota changes following PACAP treatment of mice with a human gut microbiota suffering from subacute ileitis. Subacute ileitis was induced by *T. gondii* infection of mice harboring a human gut microbiota (day 0). Starting 3 days post-infection (p.i.), mice were either treated with PACAP or placebo (PLC). Uninfected mice with a human microbiota served as control animals (Naive). At day 9 p.i., the microbiota composition of the ileal lumen **(A-H)** was determined by quantitative Real-Time PCR amplifying bacterial 16S rRNA variable regions of the main intestinal bacterial groups (expressed as 16S rRNA gene numbers per ng DNA) including the total eubacterial load as indicated. Box plots represent the 75 and 25th percentiles of the median (black bar inside the boxes). The total range and significance levels determined by one-way ANOVA test followed by Tukey post-correction test for multiple comparisons are shown. Total numbers of analyzed animals are given in parentheses. Data were pooled from four independent experiments.

Given that intestinal inflammatory conditions are accompanied by shifts in commensal gut microbiota composition of mice and men termed dysbiosis (15, 19, 26, 28–30), we quantitatively surveyed the main gut bacterial groups during subacute ileitis development in PACAP and PLC treated mice applying culture-independent 16S rRNA based methods (Figures 5A-H). Irrespective of the treatment regimen, the total eubacterial loads in the ileal lumen slightly declined until day 9 p.i. (Figure 5A). Conversely, ileitis development was accompanied by higher gene numbers of enterobacteria and enterococci in the ilea of PLC, but not PACAP mice (Figures 5B,C), whereas lactobacilli loads were higher in PACAP treated mice at day 9 p.i. as compared to both, *T. gondii* infected PLC treated mice and naive control animals (Figure 5D). Remarkably, bifidobacteria were only marginally abundant in mice suffering from subacute ileitis (Figure 5E), with a trend toward higher loads in PACAP vs. PLC mice at day 9 p.i. (Figure 5E). Furthermore, *Clostridium coccoides* gene numbers were lower in the ileum derived from *T. gondii* infected mice of either cohort (Figure 5G), whereas this was the case for *Clostridium leptum* in PLC mice (Figure 5H), but not PACAP treated counterparts (Figure 5H). Hence, subacute ileitis development in hma mice was accompanied with distinct shifts in the microbiota composition of the inflamed ileum, but to a lesser extent upon PACAP treatment.

### Extra-intestinal Anti-inflammatory Effects in PACAP Treated Mice With a Human Gut Microbiota Suffering From Subacute Ileitis

**Figure 6:**
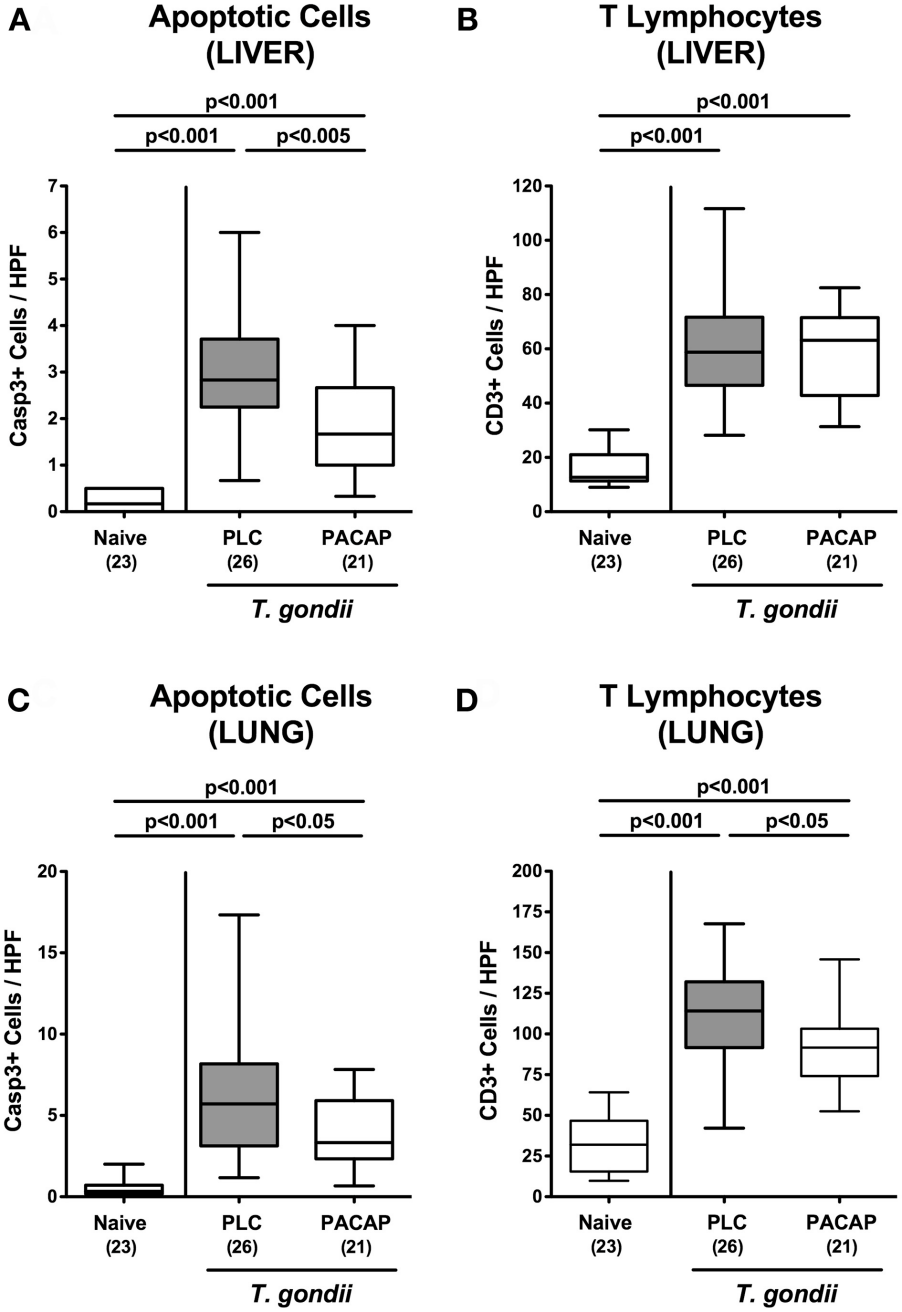
Apoptotic epithelial cell and T lymphocyte responses in extra-intestinal compartments following PACAP treatment of mice with a human gut microbiota suffering from subacute ileitis. Subacute ileitis was induced by *T. gondii* infection of mice harboring a human gut microbiota (day 0). Starting 3 days post-infection (p.i.), mice were either treated with PACAP or placebo (PLC). Uninfected mice with a human microbiota served as control animals (Naive). At day 9 p.i., the average numbers of apoptotic epithelial cells [Casp3+; **(A,C)]** and of T lymphocytes [CD3+; **(B,D)**] in at least six high power fields (HPF) were quantitatively assessed in paraffin sections of *ex vivo* biopsies derived from liver **(A,B)** and lung **(C,D)** applying *in situ* immunhistochemistry. Box plots represent the 75 and 25th percentiles of the median (black bar inside the boxes). The total range and significance levels determined by one-way ANOVA test followed by Tukey post-correction test for multiple comparisons are shown. Total numbers of analyzed animals are given in parentheses. Data were pooled from four independent experiments.

We next addressed whether anti-inflammatory effects upon PACAP treatment of hma mice with subacute ileitis were restricted to the intestinal tract or might also be observed in extra-intestinal including systemic compartments. Nine days following ileitis induction multi-fold increased numbers of apoptotic cell numbers could be observed in the livers derived from PLC and PACAP treated mice as compared to naive animals (Figure 6A; Figure S4A), but with lower counts in the latter as compared to the former (Figure 6A; Figure S4A). Irrespective of the treatment regimen, increases in apoptotic hepatic cell numbers were paralleled by more than three-fold higher numbers of T lymphocytes in the livers of *T. gondii* infected as compared to naive mice (Figure 6B; Figure S4B). Furthermore, multi-fold elevated numbers of both apoptotic cells and T lymphocytes could be assessed in the lungs during subacute ileitis (Figures 6C,D; Figures S4C,D), but with lower counts in PACAP as compared to PLC treated mice at day 9 p.i. (Figures 6C,D; Figures S4C,D).

**Figure 7:**
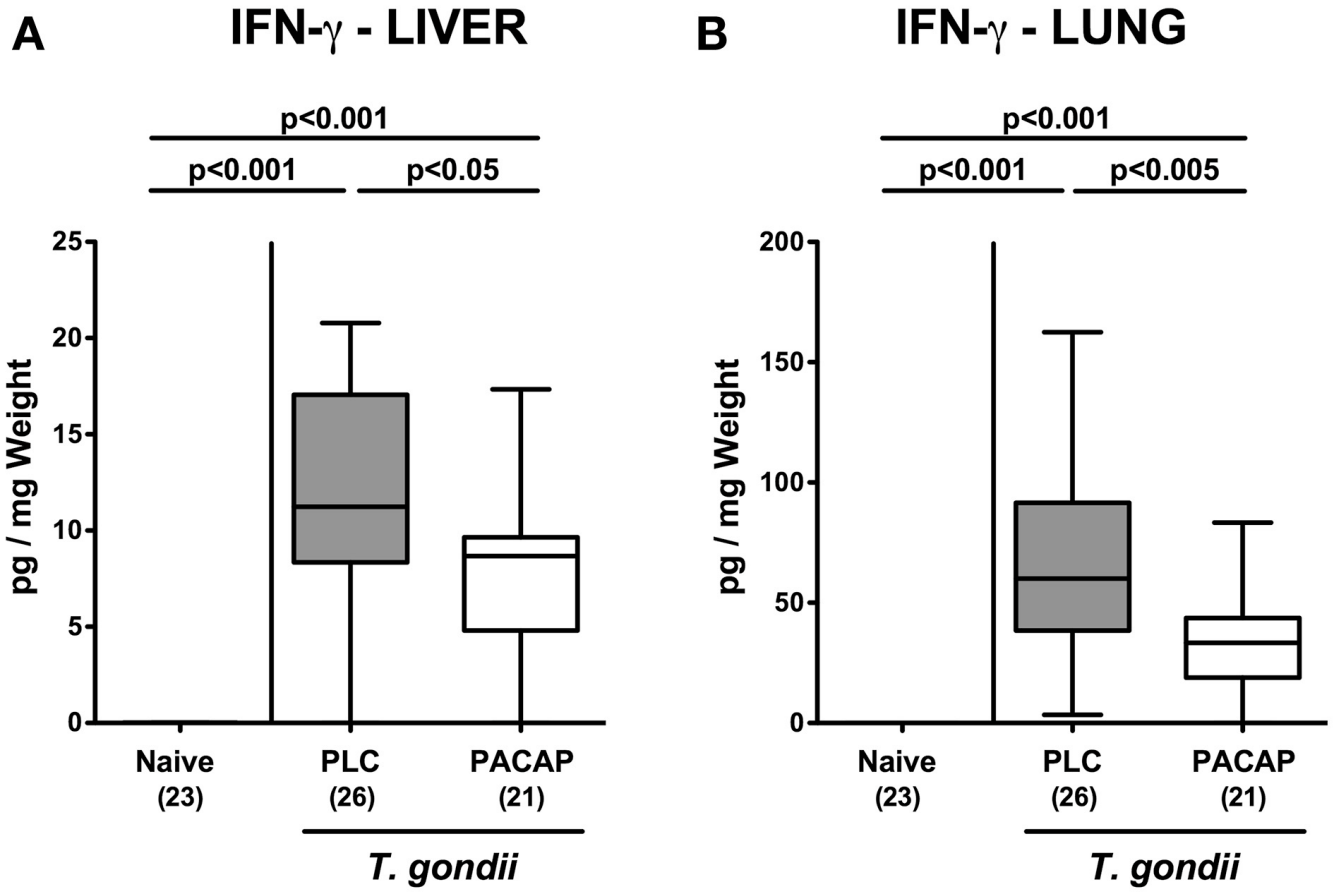
Extra-intestinal pro-inflammatory cytokine responses following PACAP treatment of mice with a human gut microbiota suffering from subacute ileitis. Subacute ileitis was induced by *T. gondii* infection of mice harboring a human gut microbiota (day 0). Starting 3 days post-infection (p.i.), mice were either treated with PACAP or placebo (PLC). Uninfected mice with a human microbiota served as control animals (Naive). At day 9 p.i., IFN-γ secretion was assessed in *ex vivo* biopsies derived from the liver **(A)** and from lung **(B)**. Box plots represent the 75 and 25th percentiles of the median (black bar inside the boxes). The total range and significance levels determined by one-way ANOVA test followed by Tukey post-correction test for multiple comparisons are shown. Total numbers of analyzed animals are given in parentheses. Data were pooled from four independent experiments.

**Figure 8:**
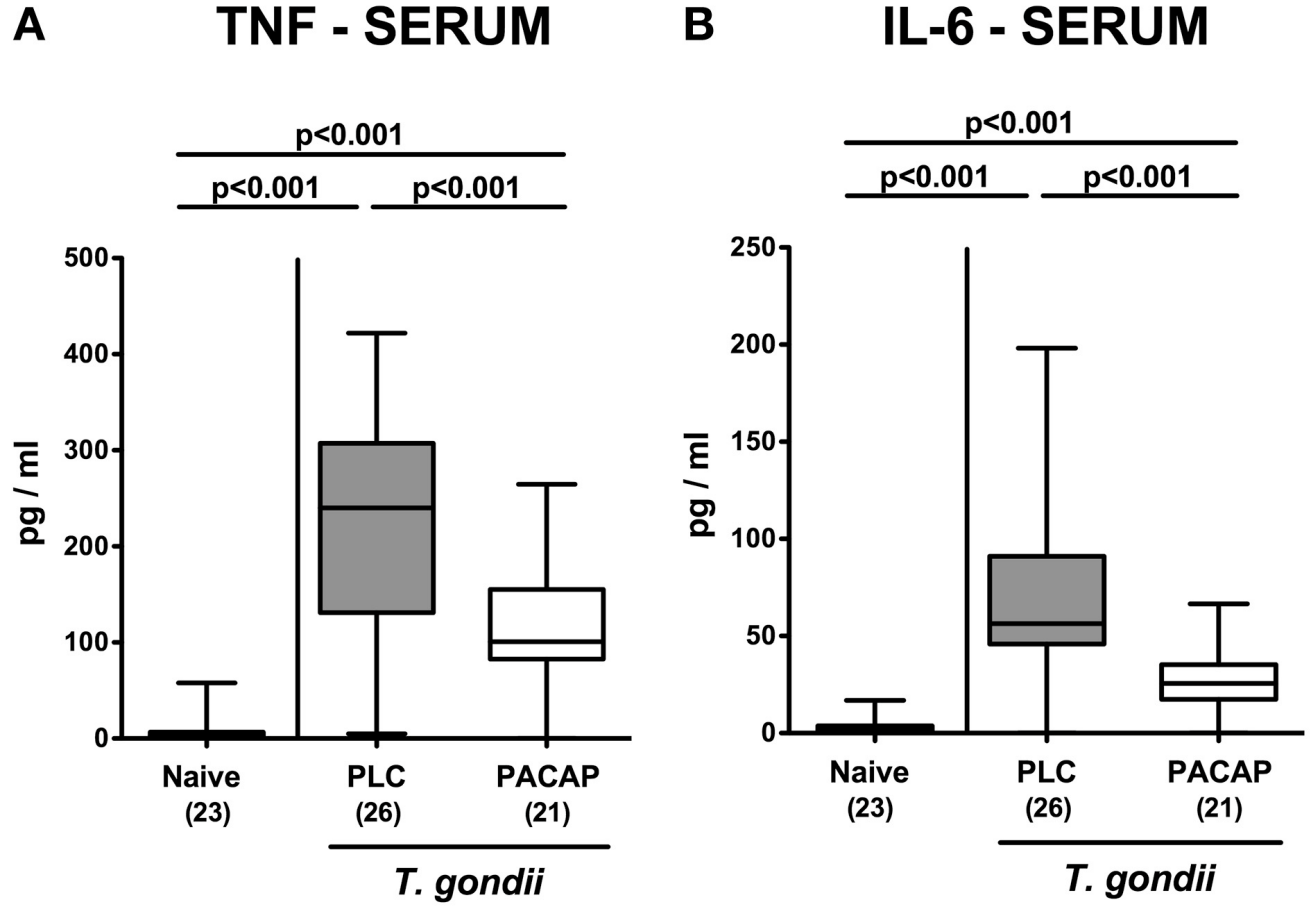
Systemic pro-inflammatory cytokine responses following PACAP treatment of mice with a human gut microbiota suffering from subacute ileitis. Subacute ileitis was induced by *T. gondii* infection of mice harboring a human gut microbiota (day 0). Starting 3 days post-infection (p.i.), mice were either treated with PACAP or placebo (PLC). Uninfected mice with a human microbiota served as control animals (Naive). At day 9 p.i., secretion of pro-inflammatory cytokines such as TNF **(A)** and IL-6 **(B)** were measured in serum samples. Box plots represent the 75 and 25th percentiles of the median (black bar inside the boxes). The total range and significance levels determined by one-way ANOVA test followed by Tukey post-correction test for multiple comparisons are shown. Total numbers of analyzed animals are given in parentheses. Data were pooled from four independent experiments.

We next measured pro-inflammatory cytokine secretion in *ex vivo* biopsies derived from respective extra-intestinal compartments and detected less distinctly increased INF-γ concentrations in the liver and lungs of PACAP as compared to PLC treated mice at day 9 p.i. (Figure 7). As for the ileum, subacute ileitis induction was further accompanied by elevated systemic concentrations of pro-inflammatory cytokines such as TNF and IL-6 (Figure 8). Strikingly, PACAP treatment resulted in ~50% lower TNF and IL-6 concentrations measured in serum samples taken at day 9 p.i. as compared to PLC control mice (Figure 8). Hence, the profound anti-inflammatory effects exerted by PACAP treatment of hma mice during subacute ileitis were not restricted to the intestinal tract, but could also be observed in extra-intestinal and even systemic compartments.

## Discussion

Several *in vitro* and *in vivo* studies revealed that PACAP exerts its neuroprotective properties via immune-modulatory and anti-apoptotic mechanisms (5, 6). Given its virtual ubiquitous expression, however, PACAP rather acts as a pleiotropic immune regulator and hence, also beyond the nervous system (6, 31). In fact, our previous work revealed that synthetic PACAP application starting prior acute ileitis induction (i.e., prophylactic regimen) ameliorated intestinal as well as extra-intestinal sequelae of peroral high-dose *T. gondii* infection in a time-of-treatment dependent manner (17) that is characterized by a T cell-driven pro-inflammatory cytokine storm with fatal outcome within 1 week (14, 15). In the present preclinical intervention study we provide additional insights into the inflammation-ameliorating properties of exogenous PACAP. The here applied subacute infection model following peroral low-dose *T. gondii* infection of mice with a human gut microbiota has been established by our group very recently (20) and is characterized by a non-lethal, far less acute course of small intestinal inflammation (as compared to high-dose *T. gondii* infection), and importantly, mimics human gut microbiota conditions. The low-dose *T. gondii* infection model of hma mice thus provides valuable opportunities to further dissect the molecular mechanisms underlying the interactions between pathogen, host immunity, and human gut microbiota during small intestinal inflammation (20).

In our present work we demonstrate that PACAP applied in a therapeutic regimen (starting post ileitis induction) exerts anti-inflammatory effects during subacute ileitis of (with respect to their gut microbiota) “humanized” mice as indicated by (1) better clinical / macroscopic conditions (including lack of body weight loss and of shrinkage of the intestinal lengths during ileitis development), (2) less distinct histopathological changes in the terminal ileum, (3) lower numbers of apoptotic epithelial cells and of T lymphocytes in both, ileum and colon, (4) less intestinal secretion of pro-inflammatory cytokines such as TNF and IL-6, (5) less apoptosis in extra-intestinal organs such as liver and lungs that is accompanied by (6) lower pulmonal T cell numbers, and (7) less IFN-γ secretion in liver and lungs, and, remarkably, (8) lower systemic concentrations of pro-inflammatory cytokines such as TNF and IL-6. Furthermore, (9) inflammation-associated gut microbiota shifts were less pronounced following PACAP as compared to PLC treatment.

The potent anti-inflammatory effects of exogenous PACAP within the intestinal tract as assessed in our actual and previous (17) study are further supported by two previous reports demonstrating that PACAP^−/−^ mice were suffering from more severe DSS-induced colitis as compared to wildtype counterparts (9, 10). When synthetic PACAP was applied via the intraperitoneal route, however, the inflammatory phenotype could be alleviated (9).

Even though peroral *T. gondii* infection of susceptible mice is known to primarily affect the terminal ileum, we extended our focus to the large intestines here. We did indeed observe marked *T. gondii* induced colonic inflammatory responses as indicated by multi-fold increased numbers of apoptotic colonic epithelial cells that could be effectively lowered by therapeutic PACAP application, which also held true for T lymphocytes within the colonic mucosa and lamina propria. In previous *in vitro* studies PACAP was shown to inhibit proliferation and migration of T lymphocytes and associated pro-inflammatory cytokine release (3, 32). An *in vivo* study further revealed that PACAP treated mice suffering from acute peritonitis displayed a diminished influx of lymphocytes into the peritoneal cavity (33).

Interestingly, synthetic PACAP was not sufficient to lower *T. gondii* induced colonic pro-inflammatory cytokine secretion (not shown), whereas this was the case in *ex vivo* biopsies derived from the ileum and from MLN. Our present observations are supported by our previous reports in both acute (25, 26) and subacute (20) murine ileitis further emphasizing that *T. gondii* induced immunopathology does not exclusively affect the terminal ileum, but might also affect the large intestines.

The well-known anti-apoptotic properties of PACAP were not restricted to the intestinal tract, but could also be assessed in extra-intestinal organs such as the liver and lungs and were paralleled by decreased IFN-γ secretion in respective organs. In line, PACAP could exert potent protective effects in human hepatocytes *in vitro* and in a murine model of liver ischemia and oxidative stress (34, 35) as well as in endotoxin-induced acute lung inflammation (36). Notably, synthetic PACAP analogs have been developed for the treatment of bronchial asthma given the anti-inflammatory and broncho-relaxant properties of the neuropeptide (36, 37).

We further addressed whether the observed inflammation-alleviating responses in PACAP treated mice were paralleled by less translocation of viable bacteria originating from the commensal gut microbiota to extra-intestinal including systemic compartments due to less distinct epithelial barrier damage. It is well-known that during acute ileitis bacterial commensals including *Escherichia coli* overgrowing the ileal lumen might migrate through the compromised intestinal epithelial barrier (i.e., “leaky gut”), come in close contact to immune cells residing in the lamina propria which might subsequently result in an exacerbation of the inflammatory immune responses (38–40). Interestingly, in our actual study bacteria could neither be cultured from MLN, nor from extra-intestinal compartments including liver, lungs and cardiac blood. Our recent study, however, revealed that mean commensal bacterial translocation rates of more than 80% could be assessed in liver and lungs during lethal acute ileitis (25, 26).

One might argue that the better outcome observed in PACAP treated hma mice with *T. gondii* induced ileitis was due to direct anti-microbial effects of the applied synthetic compound, given that both anti-parasitic (directed against *Trypanosoma brucei*) (41) and anti-bacterial (42) effects have been reported recently. In both our previous and actual studies we could, however, exclude any anti-microbial effects of the working solutions containing synthetic PACAP *in vitro* (17). Furthermore, the start of exogenous PACAP 3 days after *T. gondii* infection, in addition to comparable parasitic DNA loads as assessed in ileal *ex vivo* biopsies argue against a biological relevant anti-microbial effect of PACAP.

Meanwhile it is well-established that the orchestrated mutualistic microbiota-host relationships are of utmost importance for host cell physiology, immune homeostasis and resistance to disease (21, 43). Perturbations within the complex microbial ecosystem in the gastrointestinal tract are associated with increased susceptibility of the host to distinct intestinal morbidities including IBD, irritable bowel syndrome and coeliac disease (21, 44, 45). Likewise, intestinal inflammatory conditions are paralleled by shifts in the intestinal microbiota composition (15, 25, 26, 28–30, 46, 47). This phenomenon could also be observed in our actual survey of the microbiota composition within the inflamed ileal lumen, given that subacute ileitis development was associated with increases in enterobacteria (including *E. coli*) and enterococci, whereas obligate anaerobic *Clostridium coccoides* and *Clostridium leptum* gene numbers decreased until day 9 p.i. These microbiota shifts are supported by our previous results obtained from lethal acute ileitis of mice, but with a conventional (i.e., murine) gut microbiota (15, 46, 47). Remarkably, neither shifts toward increased enterobacteria and enterococci, nor to decreased numbers of *Clostridium leptum* could be assessed in the ilea of PACAP treated mice. Furthermore, PACAP treatment was associated with higher abundances of (potentially probiotic) lactobacilli that had been reduced during acute ileitis (15).

Interestingly, PACAP could inhibit TLR-4 activation in a model of traumatic brain injury (48). Given that *T. gondii* induced ileitis is initiated and further worsened by TLR-4 dependent signaling of bacterial LPS originating from the cell walls of Gram-negative commensals including enterobacteria such as *E. coli* accumulating in the inflamed ileal lumen (46, 47), alleviation of the TLR-4 dependent scenario constitutes a mechanistic corner stone of the multi-facetted “health-beneficial actions” of PACAP treatment.

Strikingly, exogenous PACAP could not only sufficiently dampen ileal, but also serum TNF and IL-6 concentrations, pointing toward potent systemic anti-inflammatory properties of the synthetic compound, which could also be observed in lethal acute ileitis (17). These systemic effects of exogenous PACAP are supported by several studies where PACAP could efficiently prevent from experimental endotoxin sepsis and shock (49–51).

Given the time-of-treatment dependent anti-inflammatory effect of exogenous PACAP observed during acute ileitis (17), one might further argue that starting PACAP application to hma mice before subacute ileitis induction (i.e., prophylactic regimen) might yield even more pronounced anti-apoptotic and anti-inflammatory effects in intestinal and extra-intestinal including systemic compartments. This should be addressed in future studies.

In summary, our preclinical intervention study provides strong evidence that synthetic PACAP alleviates subacute ileitis and extra-intestinal including systemic sequelae of T cell-driven immunopathology. We conclude that synthetic PACAP might open novel options for the (adjunct) therapy and/or prophylaxis of intestinal inflammation including IBD and further supports the pathophysiological role of the gut-brain axis.

## Ethics Statement

After approval of the protocols by the commission for animal experiments headed by the “Landesamt für Gesundheit und Soziales” (LaGeSo, Berlin; registration numbers G0368/11 and G0039/15), the mouse experiments were performed according to the European Guidelines for animal welfare (2010/63/EU). Animal welfare was monitored daily by assessment of clinical conditions and weight loss of mice. Mice with body weight loss of more than 20% were euthanized by cervical dislocation in accordance with the guidelines of the local authorities.

## Author Contributions

SB provided advice in design and performance of experiments, co-wrote paper. UE and AG performed experiments, analyzed data, co-edited paper. AK analyzed data, co-edited paper. ID, AT, and DR suggested critical parameters in design of experiments, co-edited paper. MH designed and performed experiments, analyzed data, wrote paper.

### Conflict of Interest Statement

The authors declare that the research was conducted in the absence of any commercial or financial relationships that could be construed as a potential conflict of interest.

## Supplementary Material

Figure S1 Microbiota composition of human donor feces. Before human FMT of secondary abiotic mice on 3 consecutive days, the main gut bacterial groups were quantitated in human fecal donor suspensions. Applying quantitative RT-PCR analysis, the 16S rRNA of the main gut commensal species such as enterobacteria (EB), enterococci (EC), lactobacilli (LB), bifidobacteria (Bif), *Bacteroides / Prevotella* species (B/P), *Clostridium coccoides* group (Clocc), *Clostridium leptum* group (Clept), as well as the total eubacterial load (TL) were assessed (gene numbers per ng DNA). The shown data are representative for four independent experiments. Total range as well as box plots representing the 75 and 25th percentiles of the median (black bar inside the boxes) are shown.

Figure S2 Primer sequences for molecular gut microbiota analyses.

Figure S3 Representative photomicrographs illustrating apoptotic epithelial cell and T lymphocyte responses in the intestinal tract following PACAP treatment of mice with a human gut microbiota suffering from subacute ileitis. Subacute ileitis was induced by *T. gondii* infection of mice harboring a human gut microbiota (day 0). Starting 3 days post-infection (p.i.), mice were either treated with PACAP or placebo (PLC). Uninfected mice with a human microbiota served as control animals (Naive). Representative photomicrographs out of four independent experiments illustrate the average numbers of apoptotic epithelial cells (Casp3+; **A,C**) and of T lymphocytes [CD3+; **(B,D)**] in at least six high power fields (HPF) as quantitatively assessed in ileal **(A,B)** and colonic **(C,D)** paraffin sections applying *in situ* immunohistochemistry at day 9 p.i.

Figure S4 Representative photomicrographs illustrating apoptotic cell and T lymphocyte responses in extra-intestinal compartments following PACAP treatment of mice with a human gut microbiota suffering from subacute ileitis. Subacute ileitis was induced by *T. gondii* infection of mice harboring a human gut microbiota (day 0). Starting 3 days post-infection (p.i.), mice were either treated with PACAP or placebo (PLC). Uninfected mice with a human microbiota served as control animals (Naive). Representative photomicrographs out of four independent experiments illustrate the average numbers of apoptotic cells [Casp3+; **(A,C)]** and of T lymphocytes [CD3+; **(B,D)]** in at least six high power fields (HPF) as quantitatively assessed in paraffin sections of *ex vivo* biopsies derived from liver **(A,B)** and lung **(C,D)** applying *in situ* immunohistochemistry at day 9 p.i.

## Abbreviations

CFU: colony forming units
DSS: dextran sodium sulfate
FMT: fecal microbiota transplantation
H&E: hematoxylin and eosin
Hma: human microbiota associated
HPF: high power field
IBD: inflammatory bowel disease
IFN: interferon
IL: interleukin
i.p.: intraperitoneal
MLN: mesenteric lymph nodes
PACAP: Pituitary adenylate cyclase-activating polypeptide
PBS: phosphate buffered saline
p.i.: post-infection
PLC: placebo
qRT-PCR: quantitative real-time polymerase chain reaction
SPF: specific pathogen free
Th: T helper cell
TNF: tumor necrosis factor
VIP: vasoactive intestinal peptide.
