# Supplementary figures and images for: Pituitary Adenylate Cyclase-Activating Polypeptide—A Neuropeptide as Novel Treatment Option for Subacute Ileitis in Mice Harboring a Human Gut Microbiota
Source: Front Immunol. 2019 Mar 22;10:554. doi: 10.3389/fimmu.2019.00554 (PMC6438926; doi:10.3389/fimmu.2019.00554)

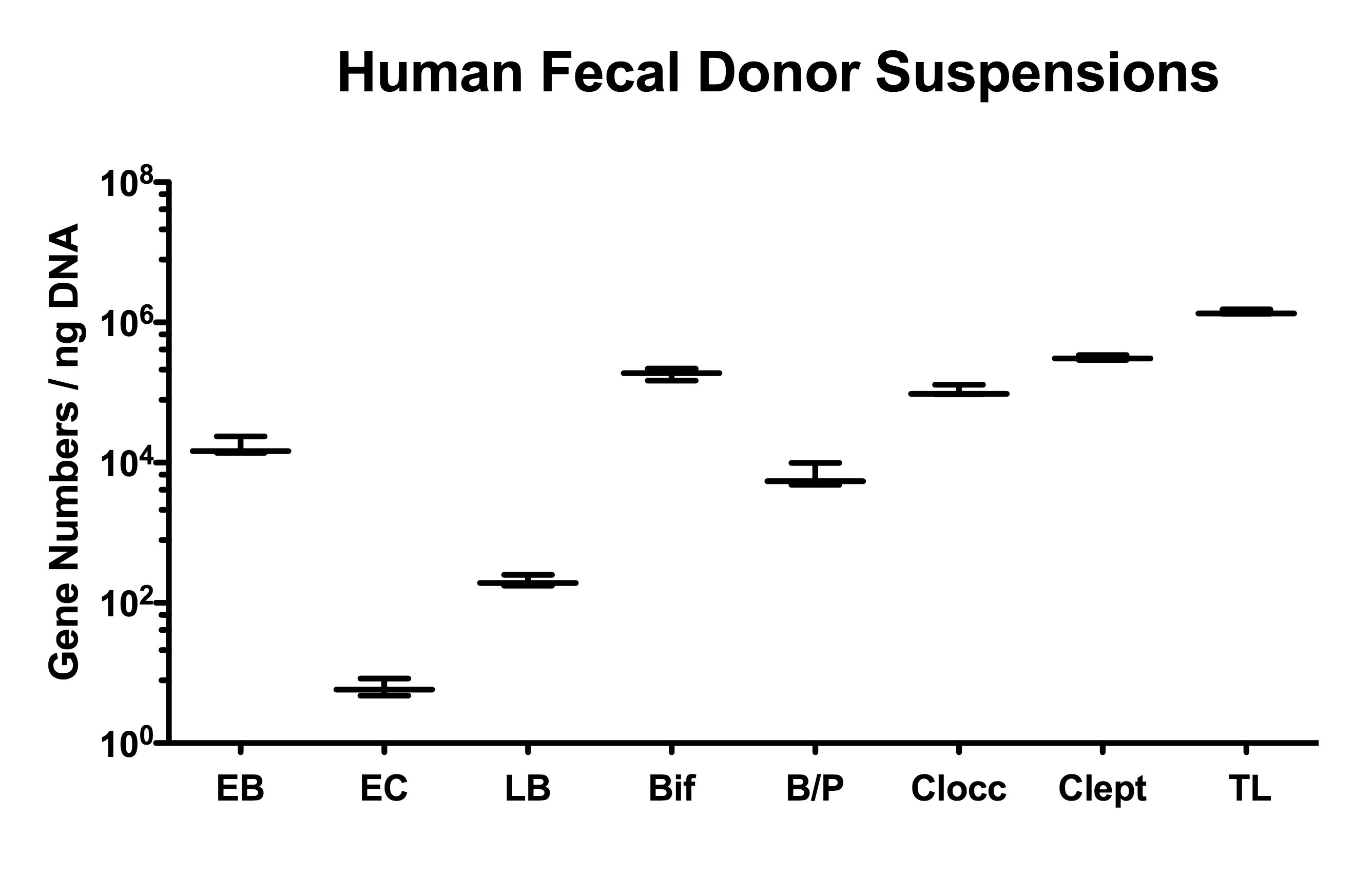

Supplement: Figure S1 — Microbiota composition of human donor feces. Before human FMT of secondary abiotic mice on 3 consecutive days, the main gut bacterial groups were quantitated in human fecal donor suspensions. Applying quantitative RT-PCR analysis, the 16S rRNA of the main gut commensal species such as enterobacteria (EB), enterococci (EC), lactobacilli (LB), bifidobacteria (Bif), Bacteroides / Prevotella species (B/P), Clostridium coccoides group (Clocc), Clostridium leptum group (Clept), as well as the total eubacterial load (TL) were assessed (gene numbers per ng DNA). The shown data are representative for four independent experiments. Total range as well as box plots representing the 75 and 25th percentiles of the median (black bar inside the boxes) are shown. [file Image_1.tiff]
