# Supplementary material for: Pituitary Adenylate Cyclase-Activating Polypeptide—A Neuropeptide as Novel Treatment Option for Subacute Ileitis in Mice Harboring a Human Gut Microbiota
Source: Front Immunol. 2019 Mar 22;10:554. doi: 10.3389/fimmu.2019.00554 (PMC6438926; doi:10.3389/fimmu.2019.00554)
Supplement: Figure S2 — Primer sequences for molecular gut microbiota analyses. [file Image_2.pdf]

| Target group | Amplicon size | Annealing | Sequence 5´-3´ | Reference |
|---|---|---|---|---|
| **Domain Bacteria (targets V3 region)** | 200 | 60°C | F: CGGYCCAGACTCCTACGGG | Lee et al. (1996) |
| | | | R: TTACCGCGGCTGCTGGCAC | |
| ***Clostridium* 16S rRNA cluster IV*** | 239 | **50°C** | F: GCACAAGCAGTGGAGT | Matsuki et al. (2004) |
| [*Clostridium leptum* subgroup) | | | R: CTTCCTCCGTTTTGTCAA | Shen et al. (2006) |
| ***Bifidobacterium* genus** | 243 | 58°C | F: TCGCGTC(C/T)GGTGTGAAAG | Rinttila et al. (2004) |
| | | | R: CCACATCCAGC(A/G)TCCAC | |
| ***Lactobacillus* group including** | 341 | 58°C | F: CACCGCTACACATGGAG | Heilig et al. (2002) |
| *Leuconostoc, Pediococcus,* | | | R: AGCAGTAGGGAATCTTCCA | Walter et al. (2001) |
| *Aerococcus, Weissella* | | | | |
| ***Mouse Intestinal Bacteroides*** | 161 | 58°C | F: CCAGCAGCCGCGGTAATA | Barman et al. (2008) |
| | | | R: CGCATTCCGCATACTTCTC | |
| ***Bacteroides* group, including** | 418 | 59°C | F: GAAGGTCCCCCACATTG | Bartosch et al. (2004) |
| *Prevotella, Porphyromonas* | | | R: CAATCGGAGTTCTTCGTG | |
| ***Enterococcus* genus** | 337 | 55 | ATCAGAGGGGGATAACACTT | Matsuda et al. (2009) |
| | | | ACTCTCATCCTTGTTCTTCTC | |
| | | | | |
| **Clostridium coccoides/** | 429 | 55°C | F: CGGTACCTGACTAAGAAGC | Rinttila et al. (2004) |
| ***Eubacterium rectale* Group** | | | R: AGTTT(C/T)ATTCTTGCGAACG | |
| | | | | |
| **gamma-Proteobacteria/Enterobacteriace** | 517 | 60°C | F: AAACTCAAATGAATTGACGG | Kühbacher et al. (2006) |
| | | | R: CTTTTGCAACCCACTCC | Sghir et al. (2000) |
