# Supplementary material for: Pituitary Adenylate Cyclase-Activating Polypeptide—A Neuropeptide as Novel Treatment Option for Subacute Ileitis in Mice Harboring a Human Gut Microbiota
Source: Front Immunol. 2019 Mar 22;10:554. doi: 10.3389/fimmu.2019.00554 (PMC6438926; doi:10.3389/fimmu.2019.00554)
Supplement: Figure S3 — Representative photomicrographs illustrating apoptotic epithelial cell and T lymphocyte responses in the intestinal tract following PACAP treatment of mice with a human gut microbiota suffering from subacute ileitis. Subacute ileitis was induced by T. gondii infection of mice harboring a human gut microbiota (day 0). Starting 3 days post-infection (p.i.), mice were either treated with PACAP or placebo (PLC). Uninfected mice with a human microbiota served as control animals (Naive). Representative photomicrographs out of four independent experiments illustrate the average numbers of apoptotic epithelial cells (Casp3+; A,C) and of T lymphocytes [CD3+; (B,D)] in at least six high power fields (HPF) as quantitatively assessed in ileal (A,B) and colonic (C,D) paraffin sections applying in situ immunohistochemistry at day 9 p.i. [file Image_3.pdf]

A

Apoptotic Cells (ILEUM)

Naive | ***T. gondii* +PLC** | ***T. gondii* +PACAP**

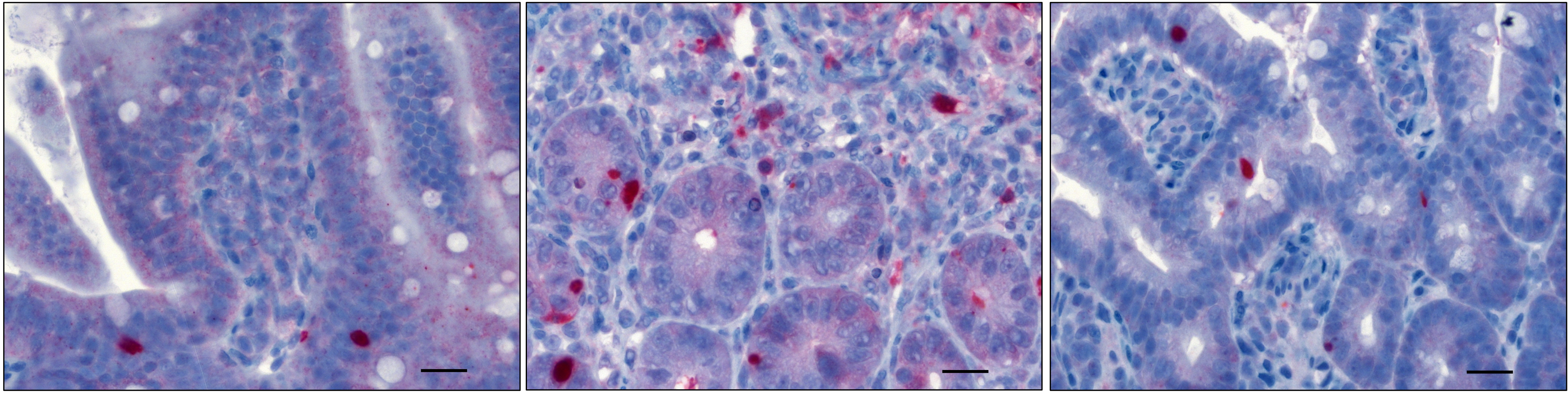

(400 x magnification, scale bar 20 µm)

B T Lymphocytes (ILEUM)

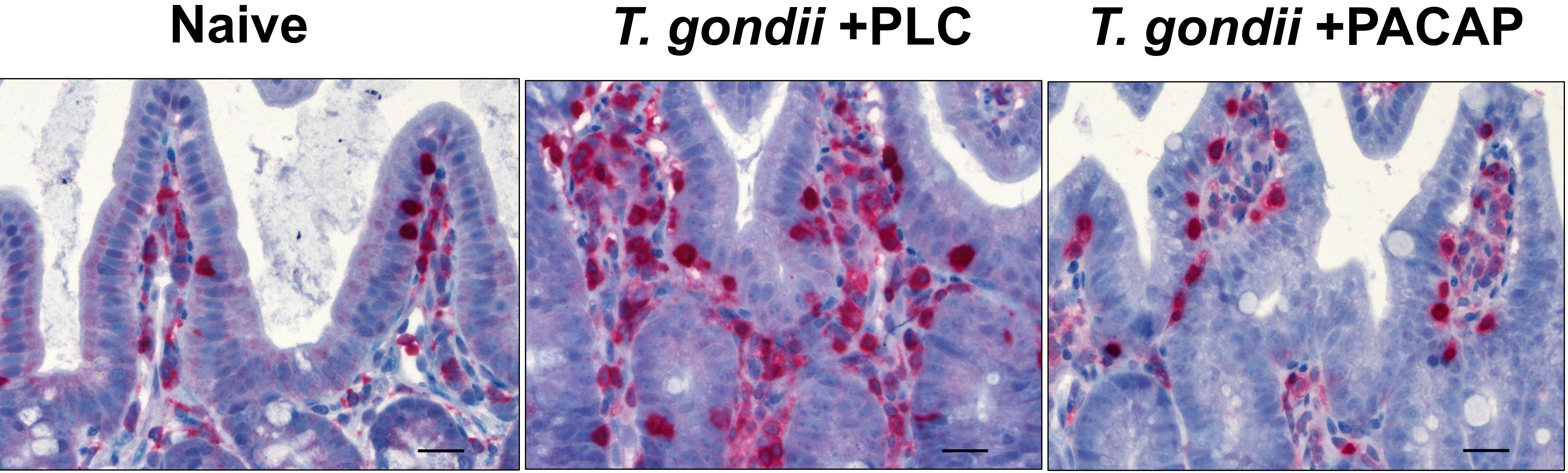

(400 x magnification, scale bar 20 µm)

C Apoptotic Cells (COLON)

Naive | ***T. gondii*** **+PLC** | ***T. gondii*** **+PACAP**

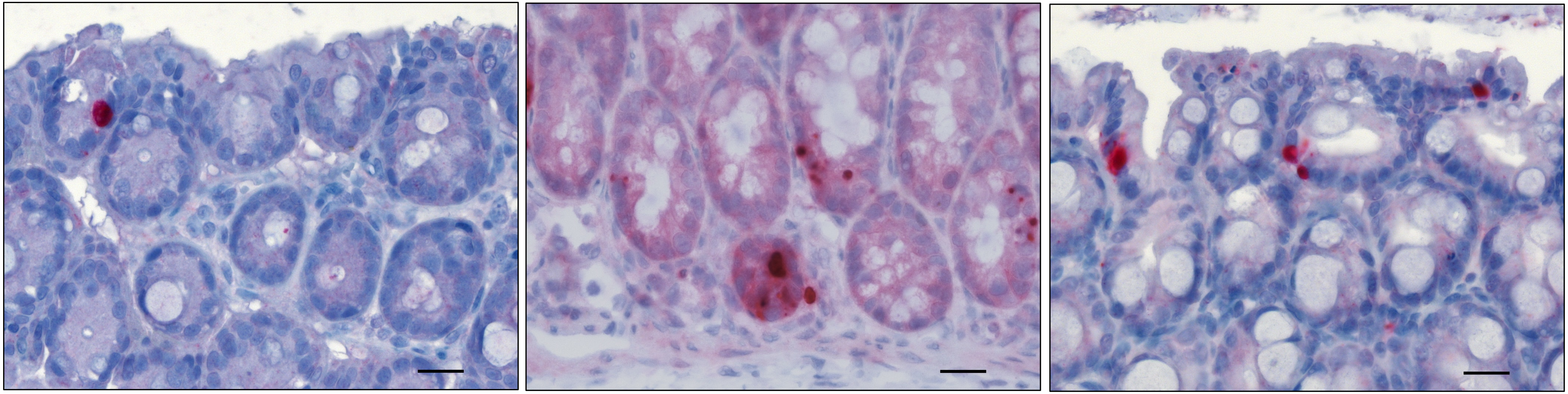

(400 x magnification, scale bar 20 µm)

D **T Lymphocytes (COLON)**

Naive ***T. gondii*** **+PLC** ***T. gondii*** **+PACAP**

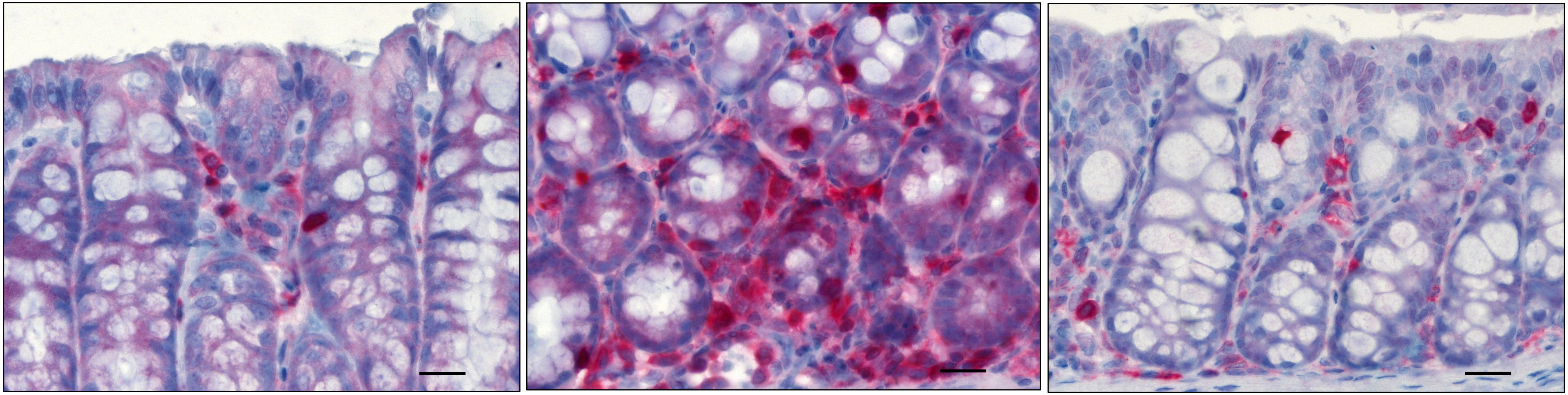

(400 x magnification, scale bar 20 µm)
