# Supplementary material for: Pituitary Adenylate Cyclase-Activating Polypeptide—A Neuropeptide as Novel Treatment Option for Subacute Ileitis in Mice Harboring a Human Gut Microbiota
Source: Front Immunol. 2019 Mar 22;10:554. doi: 10.3389/fimmu.2019.00554 (PMC6438926; doi:10.3389/fimmu.2019.00554)
Supplement: Figure S4 — Representative photomicrographs illustrating apoptotic cell and T lymphocyte responses in extra-intestinal compartments following PACAP treatment of mice with a human gut microbiota suffering from subacute ileitis. Subacute ileitis was induced by T. gondii infection of mice harboring a human gut microbiota (day 0). Starting 3 days post-infection (p.i.), mice were either treated with PACAP or placebo (PLC). Uninfected mice with a human microbiota served as control animals (Naive). Representative photomicrographs out of four independent experiments illustrate the average numbers of apoptotic cells [Casp3+; (A,C)] and of T lymphocytes [CD3+; (B,D)] in at least six high power fields (HPF) as quantitatively assessed in paraffin sections of ex vivo biopsies derived from liver (A,B) and lung (C,D) applying in situ immunohistochemistry at day 9 p.i. [file Image_4.pdf]

A Apoptotic Cells (LIVER)

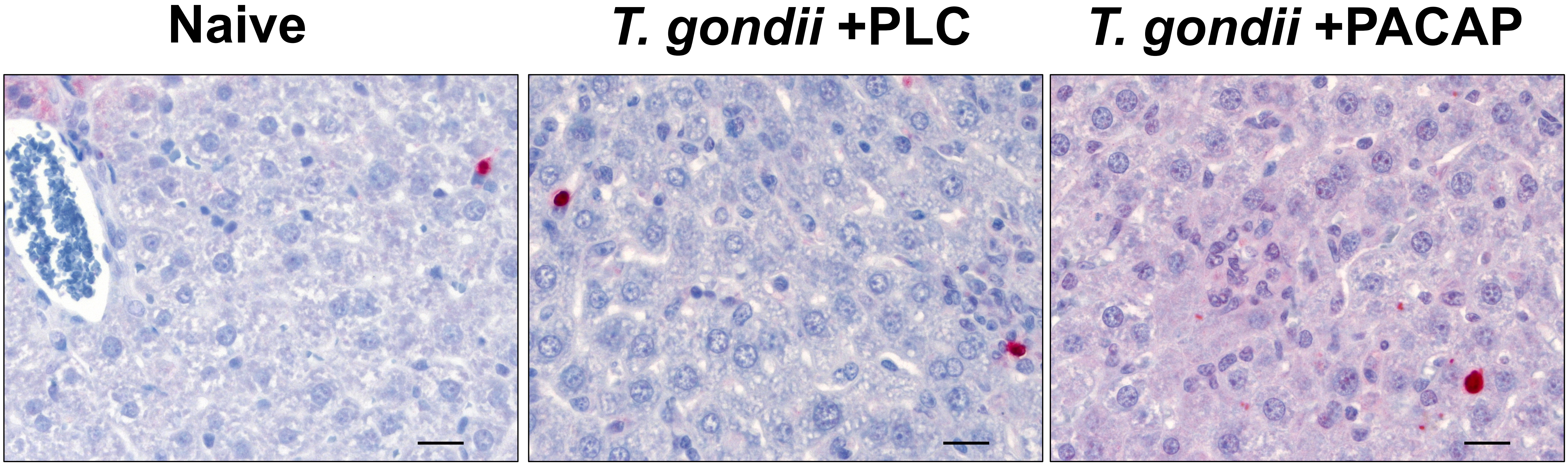

(400 x magnification, scale bar 20 µm)

B **T Lymphocytes (LIVER)**

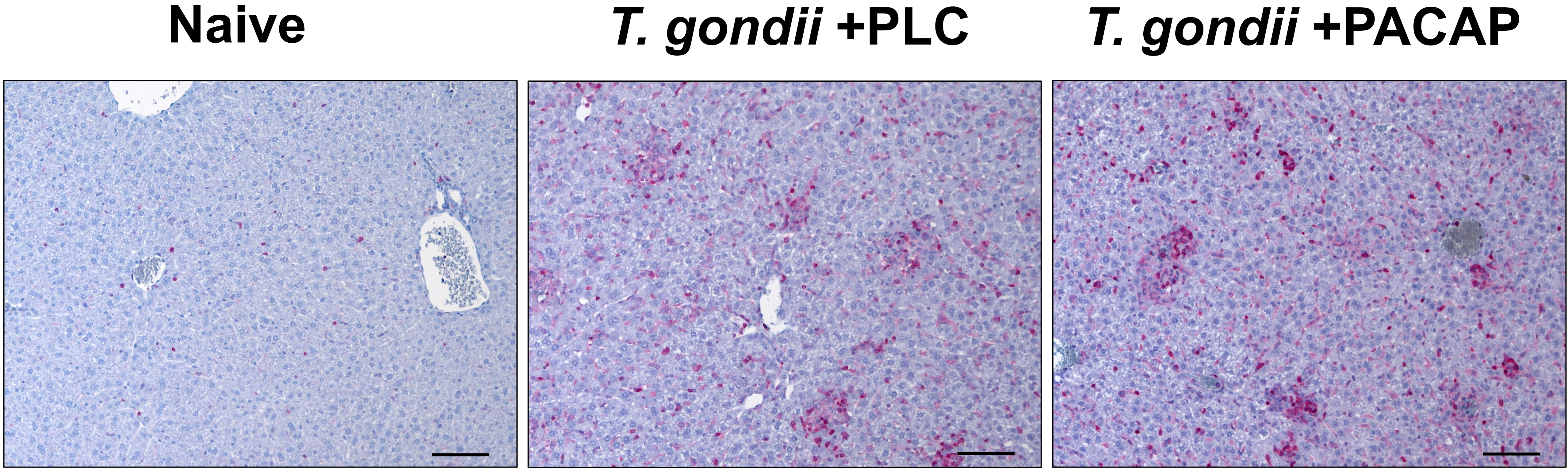

(100 x magnification, scale bar 100 µm)

C

Apoptotic Cells (LUNG)

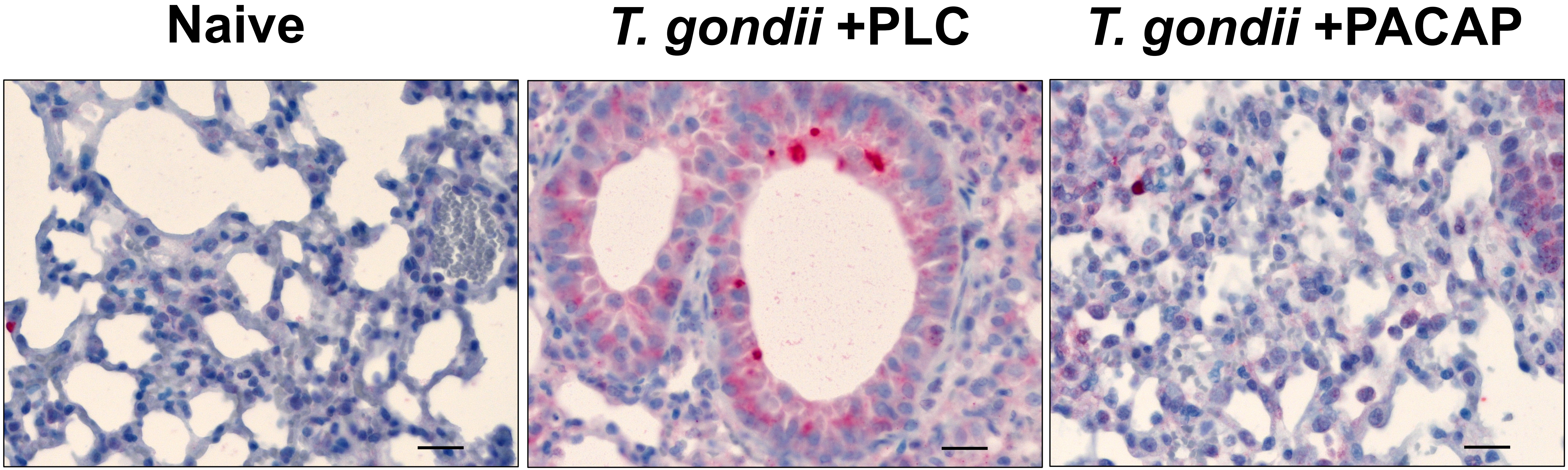

(400 x magnification, scale bar 20 µm)

D

T Lymphocytes (LUNG)

Naive | *T. gondii* +PLC | *T. gondii* +PACAP

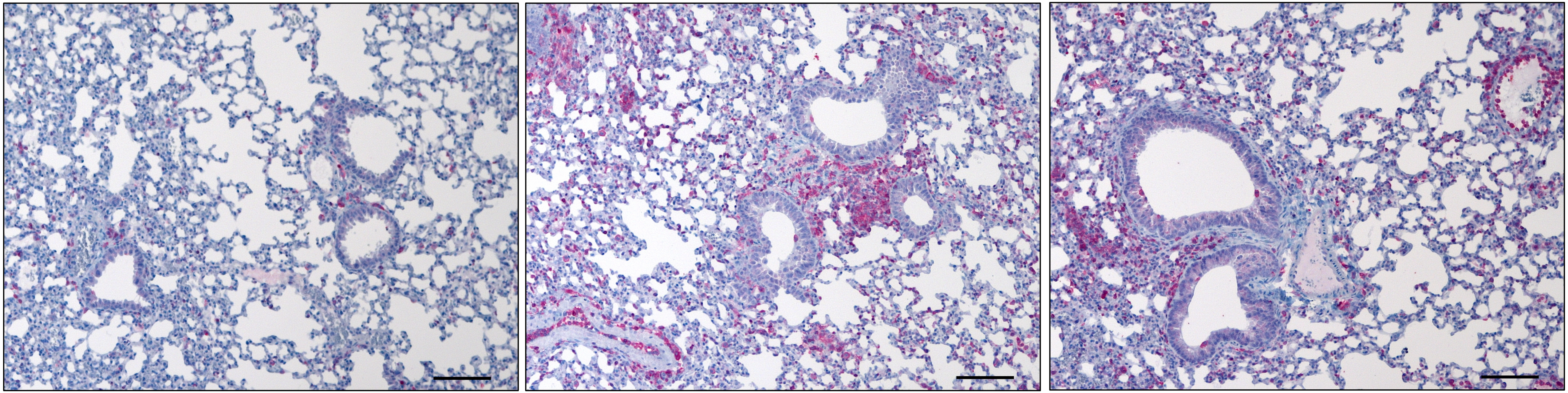

(100 x magnification, scale bar 100 µm)
